# Supplementary material for: Directions in abusive language training data, a systematic review: Garbage in, garbage out
Source: PLoS One. 2020 Dec 28;15(12):e0243300. doi: 10.1371/journal.pone.0243300 (PMC7769249; doi:10.1371/journal.pone.0243300)
Supplement: S1 Fig — (PDF) [file pone.0243300.s002.pdf]

PRISMA 2009 Flow Diagram

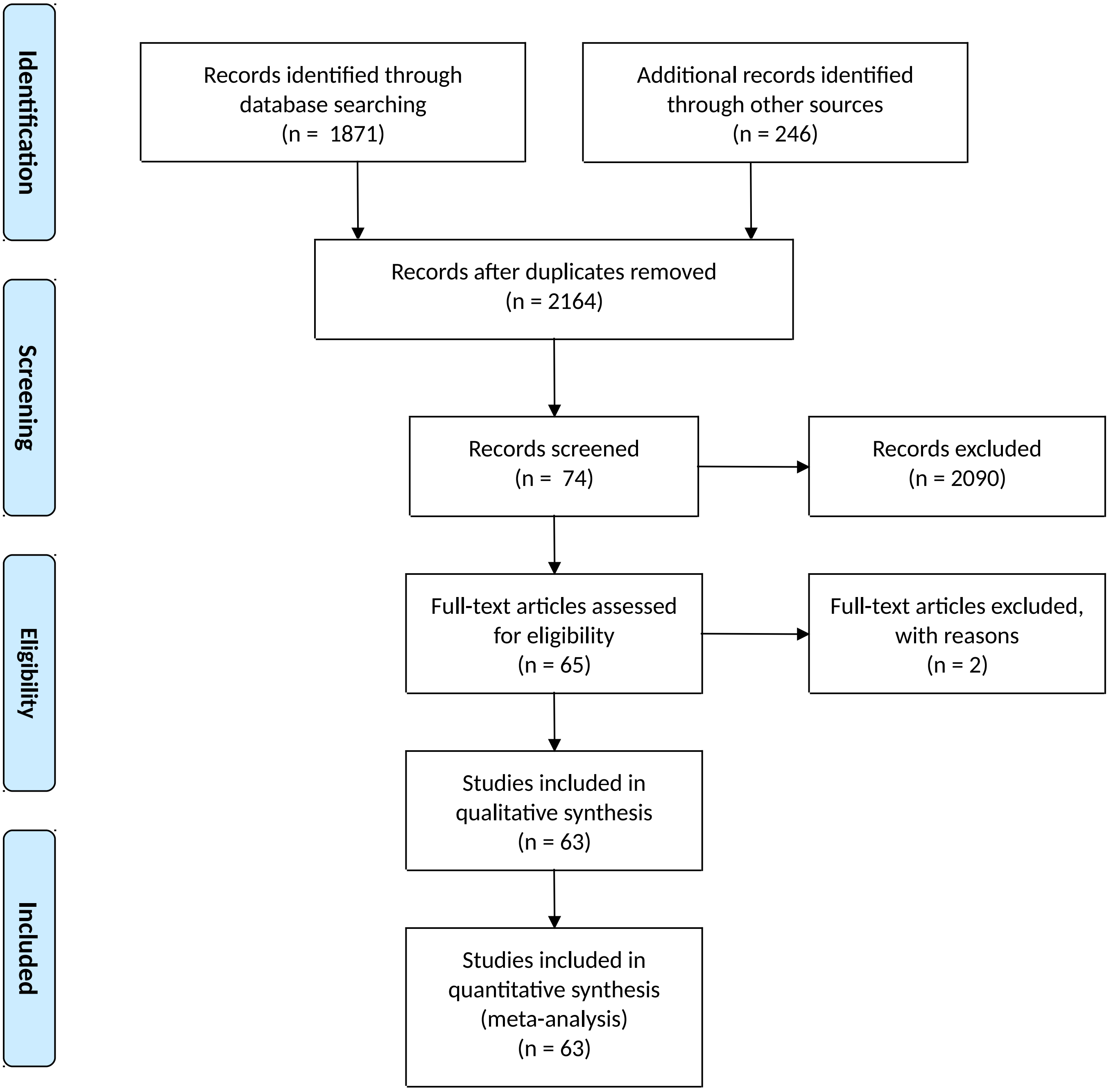

From: Moher D, Liberati A, Tetzlaff J, Altman DG, The PRISMA Group (2009). *P*referred *R*eporting *I*tems for *S*ystematic Reviews and *M*eta-*A*nalyses: The PRISMA Statement. PLoS Med 6(7): e1000097. doi:10.1371/journal.pmed1000097

For more information, visit www.prisma-statement.org.
