# Supplementary figures and images for: Directions in abusive language training data, a systematic review: Garbage in, garbage out
Source: PLoS One. 2020 Dec 28;15(12):e0243300. doi: 10.1371/journal.pone.0243300 (PMC7769249; doi:10.1371/journal.pone.0243300)

## Year of training datasets

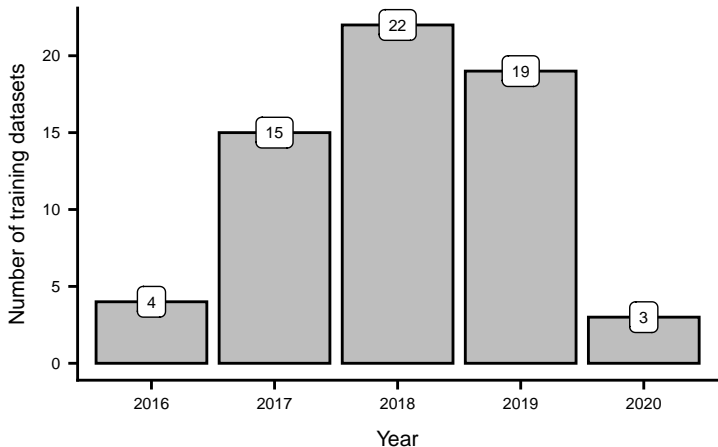

Supplement: S2 Fig — (PDF) [file pone.0243300.s003.pdf]

# Language of training datasets

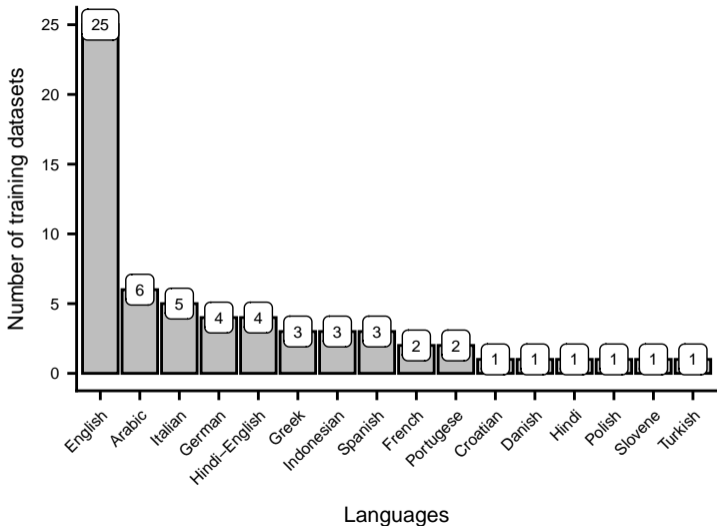

Supplement: S3 Fig — (PDF) [file pone.0243300.s004.pdf]

# Source of data of training datasets

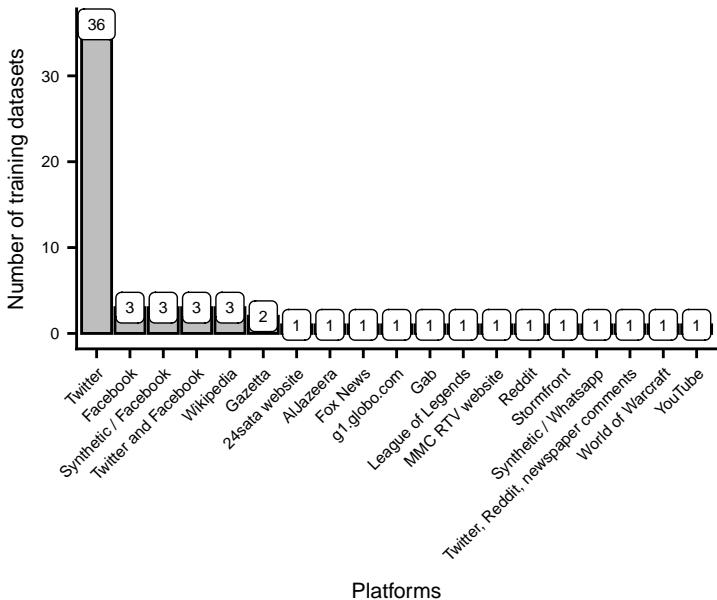

Supplement: S4 Fig — (PDF) [file pone.0243300.s005.pdf]

## Size of training datasets

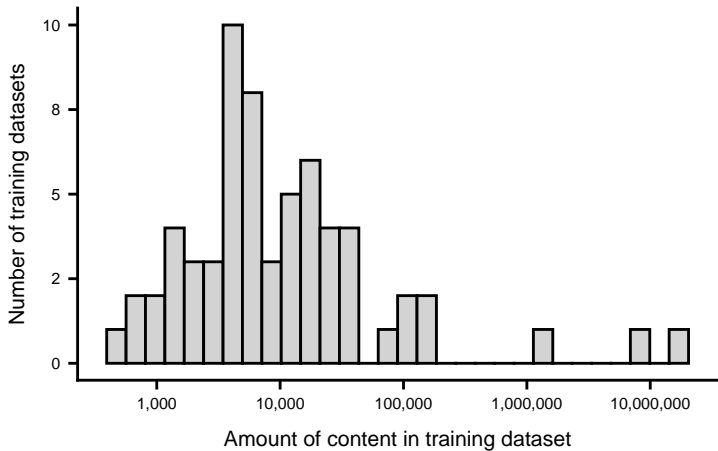

Supplement: S5 Fig — (PDF) [file pone.0243300.s006.pdf]

## Class distribution of training datasets

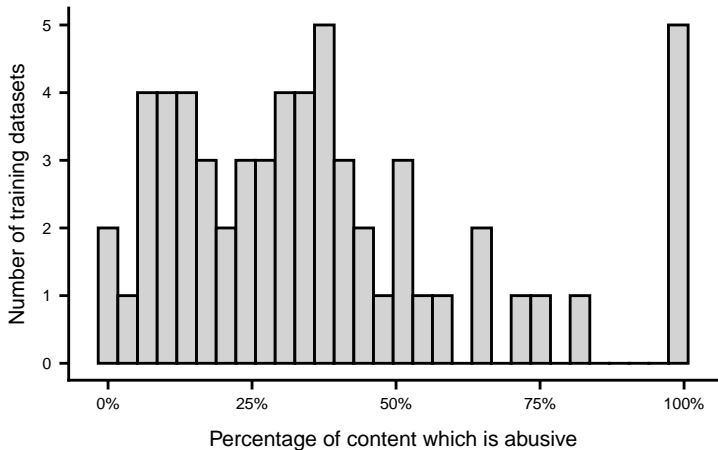

Supplement: S6 Fig — (PDF) [file pone.0243300.s007.pdf]
