# Supplementary material for: Directions in abusive language training data, a systematic review: Garbage in, garbage out
Source: PLoS One. 2020 Dec 28;15(12):e0243300. doi: 10.1371/journal.pone.0243300 (PMC7769249; doi:10.1371/journal.pone.0243300)
Supplement: S1 Appendix — [143]. (PDF) [file pone.0243300.s008.pdf]

Term	Reason
Bile	Too many medical papers
Dangerous	Too many general social issues, like problem drinking etc.
Harm	Too many other forms of online harm, like gambling etc.
Discrimination	Used in economics to describe markets and computer science to describe model performance
Attack	Very broad. Adds about 50,000+ papers.
Aggression	Too much social research. Adds about 7,000+ papers.
Stalking	Too much non-computational research.
Violence	Too much social research on violence. Adds 5,000+ papers.
Terrorist	Too much irrelevant content.
Extremist	Too much irrelevant content.
Malicious	Too much computer science technical material.
Spam	Too much computer science technical material.

Table 1. Relevant terms excluded from Scopus search

S1 Appendix. Survey search sources and keywords

SCOPUS: A search for relevant papers (shown below) was executed on 18th February 2019 in Scopus for Abstracts, Keywords and Titles, which returned 4,387 papers. All papers from the previous four years (2019, 2018, 2017 and 2016) were included (n = 1,871). The remaining papers from previous years (n = 2,516) were filtered using a generalisation of the formula outlined by [143]:

$$\frac{C}{2(Y - Y_{ref})} \geq a \qquad (1)$$

Where Y_{ref} is a reference year providing a maximum paper age, which we set to 2016; C is a paper's citation count at time of survey; Y its year of publication; and a is a threshold coefficient, set to 1 for this search. This method is biased towards including more recent publications. From this, an additional 219 papers were added to the dataset, bringing the total to 2,090 papers. These papers were then screened by two annotators (both of whom were researchers with experience in this area of research).

The initial coding guidelines for inclusion of datasets were developed by the authors, based on prior research. First, two analysts annotated 50 papers. They jointly reviewed their annotations and updated the guidelines. This was repeated for another 50 papers. Each analyst then independently coded up all of the remaining 1,990 papers. Analysts agreed in 80% of cases. Of the remaining 20%, each paper was discussed and inclusion/exclusion agreed. The coding guidelines for inclusion were updated once more to reflect the final discussion.

The search term used for Scopus is described in the paper. Through iterative searching on Scopus, we found that several terms which are widely used in the abusive content literature are inappropriate for searching as they return too many non-relevant papers, as shown in Table 1.

SPECIALIST LITERATURE: The ACL Anthology and arXiv cs.cl category both offer automatic topical filtering, reducing the number of spurious results. We searched for a similar set of terms (described in the paper).

Searches over these two sources identified 60 datasets. Solicited recommendations identified a further 27 datasets. Combining and de-duping these left 74 datasets. Of these, nine were initially screened out because on closer inspection they were not relevant to the domain. A further two were excluded for not making the datasts publicly available. This leaves 63 datasets which were analysed in the paper.
