# Supplementary material for: Directions in abusive language training data, a systematic review: Garbage in, garbage out
Source: PLoS One. 2020 Dec 28;15(12):e0243300. doi: 10.1371/journal.pone.0243300 (PMC7769249; doi:10.1371/journal.pone.0243300)
Supplement: S2 Appendix — (PDF) [file pone.0243300.s009.pdf]

S2 Appendix. Datasets included in survey
The datasets surveyed in this paper are summarized in Table 2.

Short Title	Year	Language	Level	Source	Medium	Task	Annotation	Size	Abuse %	Access to dataset
Datasets of Slovene and Croatian Moderated News Comments (Slovene)	2018	Slovene	Posts	MMC RTV website	Text	Binary	Experts, professional	7600000	0.08	Public (encrypted)
Datasets of Slovene and Croatian Moderated News Comments (Croatian)	2018	Croatian	Posts	24sata website	Text	Binary	Experts, professional	17000000	0.02	Public (encrypted)
Automated Hate Speech Detection and the Problem of Offensive Language	2017	English	Posts	Twitter	Text	Binary	Crowdsourced	24802	0.06	Public
Hate Speech Dataset from a White Supremacy Forum	2018	English	Posts	Stormfront	Text	Multiple	Experts, academic	9916	0.11	Public
Hateful Symbols or Hateful People?	2016	English	Posts	Twitter	Text	Multiple	Experts, academic	16914	0.32	Public (dehydrated)
A Quality Type-aware Annotated Corpus and Lexicon for Harassment Research	2018	English	Posts	Twitter	Text	Multiple	Experts, academic	24189	0.13	Access on request
An Italian Twitter Corpus of and Hate Speech against Immigrants	2018	Italian	Posts	Twitter	Text	Multiple	Mix	1827	0.13	Public (dehydrated)
Measuring the Reliability of Hate Speech Annotations	2017	German	Posts	Twitter	Text	Binary	Experts, academic	469	NA	Public
Detecting Online Hate Speech Using Context Aware Models	2018	English	Posts	Fox News	Text	Binary	Experts, academic	1528	0.28	Public
Are You a Racist or Am I Seeing Things?	2016	English	Posts	Twitter	Text	Multiple	Mix	4033	0.16	Public (dehydrated)
Large Scale Crowdsourcing and Characterization of Twitter Abusive Behavior	2018	English	Posts	Twitter	Text	Multiple	Crowdsourced	80000	0.18	Public (dehydrated)
A Large Labeled Corpus for Online Harassment Research	2017	English	Posts	Twitter	Text	Binary	Crowdsourced	35000	0.16	Access on request
Creating a WhatsApp Dataset to Study Pre-teen Cyberbullying	2018	Italian	Posts	Synthetic / Whatsapp	Text	Binary	Synthetic	14600	0.08	Public
Aggression-annotated Corpus of Hindi-English Code-mixed Data (Facebook)	2018	Hindi-English	Posts	Facebook	Text	Multiple	Experts, academic	18000	0.06	Access on request
Aggression-annotated Corpus of Hindi-English Code-mixed Data (Twitter)	2018	Hindi-English	Posts	Twitter	Text	Multiple	Experts, academic	21000	0.27	Access on request
When Does a Compliment Become Sexist?	2017	English	Posts	Twitter	Text	Multiple	Experts, academic	712	1	Public (dehydrated)
Did You Offend Me? Classification of Offensive Tweets in Hinglish Language	2018	Hindi-English	Posts	Twitter	Text	Multiple	Experts, academic	3189	0.65	Public
Analysis and Detection of Religious Hate Speech in the Arabic Twittersphere	2018	Arabic	Posts	Twitter	Text	Binary	Crowdsourced	6136	0.45	Public (dehydrated)
A Dataset of Hindi-English Code-Mixed Social Media Text for Hate Speech Detection	2018	Hindi-English	Posts	Twitter	Text	Binary	Experts, academic	4575	0.36	Public (dehydrated)
Deep Learning for User Comment Moderation Flagged Comments	2017	Greek	Posts	Gazetta	text	Binary	Experts, professional	1450000	0.34	Public
Deep Learning for User Comment Moderation Moderated Comments	2017	Greek	Posts	Gazetta	text	Binary	Experts, academic	1500	0.22	Public
Ex Machina: Personal Attacks Seen at Scale Personal attacks	2017	English	Posts	Wikipedia	Text	Binary	Crowdsourced	115737	0.12	Public
Ex Machina: Personal Attacks Seen at Scale Toxicity	2017	English	Posts	Wikipedia	Text	Multiple	Crowdsourced	100000	NA	Public
Ex Machina: Personal Attacks Seen at Scale Aggression and friendliness	2017	English	Posts	Wikipedia	Text	Multiple	Crowdsourced	160000	NA	Public

Table 2. Datasets surveyed (1/3) in this paper.

Short Title	Year	Language	Level	Source	Medium	Task	Annotation	Size	Abuse %	Access to dataset
Detecting Offensive Statements Towards Foreigners in Social Media	2017	German	Posts	Facebook	Text	Multiple	Experts, academic	5836	0.11	Public
Detecting cyberbullying in online communities (World of Warcraft)	2016	English	Posts	World of Warcraft	Text	Binary	Experts, academic	16975	0.01	Public
Detecting cyberbullying in online communities (League of Legends)	2016	English	Posts	League of Legends	Text	Binary	Experts, academic	17354	0.01	Public
Abusive Language Detection in Indonesian Social Media	2018	Indonesian	Posts	Twitter	Text	Multiple	Crowdsourced	2016	0.54	Public
Abusive Language Detection on Arabic Social Media (Twitter)	2017	Arabic	Posts	Twitter	Text	Multiple	Crowdsourced	1100	0.59	Public
Abusive Language Detection on Arabic Social Media (Al Jazeera)	2017	Arabic	Posts	AlJazeera	Text	Multiple	Crowdsourced	32000	0.81	Public
A Hierarchically-Labeled Portugese Hate Speech Dataset	2019	Portuguese	Posts	Twitter	Text	Multiple	Experts, academic	3059	0.32	Public (dehydrated)
Hate Speech Detection in the Indonesian Language	2017	Indonesian	Posts	Twitter	Text	Binary	Experts, academic	713	0.36	Public
Anti-Social Behaviour in Online Communication in Arabic	2018	Arabic	Posts	YouTube	Text	Binary	Experts, academic	15050	0.39	Public
L-HSAB: A Levantine Twitter Dataset for Hate Speech and Abusive Language	2019	Arabic	Posts	Twitter	Text	Multiple	Experts, academic	5846	0.38	Public
Predicting the Type and Target of Offensive Posts in Social Media	2019	English	Posts	Twitter	Text	Multiple	Mix	14100	0.33	Access on request
hatEval, SemEval-2019 Task 5 (English)	2019	English	Posts	Twitter	Text	Multiple	Mix	13000	0.4	Access on request
hatEval, SemEval-2019 Task 5 (Spanish)	2019	Spanish	Posts	Twitter	Text	Multiple	Mix	6600	0.4	Access on request
GermEval 2018	2018	German	Posts	Twitter	Text	Multiple	Experts, academic	8541	0.34	Access on request
Multi-Label Hate Speech and Abusive Language Detection in Indonesian Twitter	2019	Indonesian	Posts	Twitter	Text	Multiple	Crowdsourced	13169	0.42	Public
Offensive Comments in the Brazilian Web	2017	Portuguese	Posts	g1.globo.com	Text	Multiple	Experts, academic	1250	0.33	Public
Overview of MEX-A3T at IberEval 2018	2018	Spanish	Posts	Twitter	Text	Binary	Experts, academic	11000	0.32	Access on request
Automatic Misogyny Identification at IberEval 2018 (English)	2018	English	Posts	Twitter	Text	Multiple	Crowdsourced	3977	0.47	Access on request
Automatic Misogyny Identification at IberEval 2018 (Spanish)	2018	Spanish	Posts	Twitter	Text	Multiple	Crowdsourced	4138	0.5	Access on request
EVALITA 2018 Hate Speech Detection Task (Facebook)	2018	Italian	Posts	Facebook	Text	Multiple	Experts, academic	4000	0.51	Access on request
EVALITA 2018 Hate Speech Detection Task (Twitter)	2018	Italian	Posts	Twitter	Text	Multiple	Mix	4000	0.32	Access on request
CONAN - COunter NArratives through Nichesourcing (English)	2019	English	Posts	Synthetic / Facebook	Text	Multiple	Synthetic	1288	1	Public
CONAN - COunter NArratives through Nichesourcing (French)	2019	French	Posts	Synthetic / Facebook	Text	Multiple	Synthetic	1719	1	Public
CONAN - COunter NArratives through Nichesourcing (Italian)	2019	Italian	Posts	Synthetic / Facebook	Text	Multiple	Synthetic	1071	1	Public
Characterizing and Detecting Hateful Users on Twitter	2018	English	Users	Twitter	Text	Binary	Crowdsourced	4972	0.11	Access on request
Learning to Intervene in Online Hate Speech (Gab)	2019	English	Posts	Gab	Text	Binary	Crowdsourced	33776	0.43	Public
Learning to Intervene in Online Hate Speech (Reddit)	2019	English	posts	Reddit	Text	Binary	Crowdsourced	22324	0.24	Public
Multilingual and Multi-Aspect Hate Speech Analysis (English)	2019	English	Posts	Twitter	Text	Multiple	Crowdsourced	5647	0.76	Public

Table 2. Datasets surveyed (2/3) in this paper.

Short Title	Year	Language	Level	Source	Medium	Task	Annotation	Size	Abuse %	Access to dataset
Multilingual and Multi-Aspect Hate Speech Analysis (French)	2019	French	Posts	Twitter	Text	Multiple	Crowdsourced	4014	0.72	Public
Multilingual and Multi-Aspect Hate Speech Analysis (Arabic)	2019	Arabic	Posts	Twitter	Text	Multiple	Crowdsourced	3353	0.64	Public
Exploring Hate Speech Detection in Multimodal Publications	2019	English	Posts	Twitter	Text + Images	Multiple	Crowdsourced	149823	0.25	Public
Peer to Peer Hate: Hate Speech Instigators and Their Targets	2018	English	Posts	Twitter	Text	Binary	Crowdsourced	27330	0.98	Public
Offensive Language Identification in Greek	2020	Greek	Posts	Twitter	Text	Multiple	Experts, academic	4779	0.29	Access on request
A Corpus of Turkish Offensive Language on Social Media	2020	Turkish	Posts	Twitter	Text	Multiple	Experts, academic	36232	0.19	Access on request
Offensive Language and Hate Speech Detection for Danish	2019	Danish	Posts	Twitter, Reddit and news comments	Text	Multiple	Experts, academic	3600	0.12	Access on request
PolEval 2019 Shared Task 6	2019	Polish	Posts	Twitter	Text	Multiple	Mix	10041	0.09	Public
Overview of the HASOC track at FIRE 2019 (English)	2019	English	Posts	Twitter and Facebook	Text	Multiple	Experts, academic	7005	0.36	Public
Overview of the HASOC track at FIRE 2019 (Hindi)	2019	Hindi	Posts	Twitter and Facebook	Text	Multiple	Experts, academic	5983	0.51	Public
Overview of the HASOC track at FIRE 2019 (German)	2019	German	Posts	Twitter and Facebook	Text	Multiple	Experts, academic	4669	0.24	Public

Table 2. Datasets surveyed (3/3) in this paper.
